# Evaluation of *Trigonella foenum-graecum* extract in combination with swimming exercise compared to glibenclamide consumption on type 2 Diabetic rodents

**DOI:** 10.3402/fnr.v59.29717

**Published:** 2015-12-22

**Authors:** Sajad Arshadi, Mohammad Ali Azarbayjani, Fatemeh Hajiaghaalipour, Ashril Yusof, Maghsoud Peeri, Salar Bakhtiyari, Robert S. Stannard, Noor Azuan Abu Osman, Firouzeh Dehghan

**Affiliations:** 1 Exercise Physiology Department, Faculty of Physical Education, Islamic Azad University South Tehran Branch, Tehran, Iran; 2 Exercise Physiology Department, Islamic Azad University Central Tehran Branch, Tehran, Iran; 3 Department of Molecular Medicine, Faculty of Medicine, University of Malaya, Kuala Lumpur, Malaysia; 4 Department of Exercise Science, Sports Centre, University of Malaya, Kuala Lumpur, Malaysia; 5 Department of Clinical Biochemistry, Faculty of Medicine, Ilam University of Medical Sciences, Ilam, Iran; 6 School of Sport and Exercise, Massey University, Palmerston North, New Zealand; 7 Department of Biomedical Engineering, Faculty of Engineering, University of Malaya, Kuala Lumpur, Malaysia

**Keywords:** swimming training, fenugreek seed, glibenclamide, lipid profile, diabetic, leptin, insulin resistance

## Abstract

**Background/objective:**

The purpose of the present study was to evaluate the effect of fenugreek seed extract in combination with swimming exercise compared to glibenclamide consumption on type 2 diabetic rats.

**Design:**

The acute toxicity test was carried out to choose the safe doses and identify the toxicity effects of the fenugreek seed extract. To investigate the hypoglycemic effect of the extract and its effect in combination with swimming training, 80 Wistar Kyoto male streptozotocin-induced diabetic rats were divided randomly into eight groups: diabetic control (C); fenugreek seed extract 0.8 g/kg (F1); fenugreek extract 1.6 g/kg (F2); swimming training (S); swimming training plus fenugreek extract 0.8 g/kg (SF1); swimming training plus fenugreek extract 1.6 g/kg (SF2); glibenclamide (G) and swimming training plus glibenclamide (SG). The rats were orally administrated with the treatments once a day with the respective treatment, and the training groups were subjected to swimming training every day for 60 min. Fasting blood samples were collected to measure fasting blood glucose, lipid profile, adiponectin, leptin, and insulin concentrations.

**Results:**

The results obtained from acute toxicity study showed no toxicity effect of fenugreek seed extract on the tested dose. Biochemical analysis showed significant improvements in all of the groups compared to the control group (*p*<0.05). Plasma insulin concentration and insulin resistance (HOMA-IR) was significantly reduced in treated groups compared with the diabetic control group. Plasma leptin were significantly decreased in treated groups compared with the control group; while adiponectin had markedly increased (*p*<0.05).

**Conclusion:**

The findings suggest that fenugreek seed consuming, alongside swimming exercise, has a strong therapeutic effect on the improvement of diabetic parameters.

The global prevalence of diabetes, particularly type 2, is steadily increasing due to sedentary lifestyle, life span, obesity, and consumption of energy-rich foods and nutrient-dense foods. Diabetes is characterized by increased level of blood glucose and impaired carbohydrate, fat, and protein metabolism (1, 2). The metabolic disorder type 2 diabetes mellitus is similarly related to irregular levels of lipids and lipoproteins, including cholesterol, low-density lipoprotein (LDL), very low-density lipoprotein (VLDL), high-density lipoprotein (HDL), triglyceride (TG), as well as leptin and adiponectin (3). The possible contribution of adipocyte-derived proteins such as leptin and adiponectin has been suggested in the pathologic mechanism linking obesity and insulin resistance (4, 5). Although much effort is being expended on the prevention of diabetes, finding new treatment strategies for reduction has been the mainstay in medicine. Some of the more popular treatments for type 2 diabetes directly target circulating glucose concentrations and include pharmacological agents such as metformin, which reduces hepatic glucose production, or glibenclamide, which increases pancreatic insulin release. Glibenclamide, known as one of the oral antidiabetics in the World Health Organization Model List of Essential Medicines, is used in type 2 diabetes treatment (6).

With respect to the side effects of these pharmacological drugs, natural products have attracted attention as source materials for the development of alternative therapy or new antidiabetic drugs for the control of diabetes complications. In fact, plants have been used to cure diabetes mellitus in the ancient times in Egypt (7). Recently, studies have suggested the antidiabetic potential of herbal medicine through their modulatory effects on glucose transporter by interfering in different signaling pathways (8). To disclose the action of antidiabetic plants’ potential, discussing systematic and scientific points is necessary for using these plants as antidiabetic agents (9).

Fenugreek (*Trigonella foenum graecum*) is an annual herb, belonging to the Leguminosae family, native to Southeastern Europe, Northern Africa, and Central Asia (10). Fenugreek seed is known to be a medicinal plant, originating in India and Northern Africa, and it has been used traditionally for its two main pharmacological properties that of being antidiabetic and hypocholesterolaemic (11–13). The hypoglycemic effect of fenugreek seed (family Leguminosae) has been investigated in various animal models (14, 15), as well as in insulin-dependent diabetes mellitus (IDDM) patients (16) and non-insulin-dependent diabetes mellitus (NIDDM) patients (17). In addition to its antidiabetic property, the antioxidant activity of the seeds also has been shown in many studies (12, 18, 19). Therefore, it could potentially be a valuable candidate in the control of blood sugar in untreated diabetes.

Regular physical activity has also been shown to be effective in maintaining glucose homeostasis. Aerobic exercise is generally prescribed to diabetic patients, as it treats associated glucose, lipid, and cardiovascular abnormalities, as well as aid in weight loss and maintenance (20). The accumulation of intramuscular lipid in sedentary people is associated with insulin resistance. Lipoprotein lipase (LPL) activity in skeletal muscles increases during heavy exercise activity, which is effective in reducing intramyocellular lipid (IMCL) accumulation (21). Leptin and Adiponectin, which are two major hormones produced by adipose tissue and associated with type 2 diabetes, normalize insulin action (22). Circulating concentrations of leptin have been shown to decrease after endurance exercise training in diabetic subjects (23) or healthy controls (24). Other than adiposity, insulin is one of the factors that regulate the plasma leptin concentration (25). The association of plasma leptin concentrations with satiety is well known (26), and this leptin stimulates lipidsoxidation (27) and increases expenditure of energy (28). These effects suggest that leptin plays a crucial role in homeostasis of energy and helps to limit energy storage in adipose tissue of mammals.

The different therapeutic effects of fenugreek seed extract and physical activity have been investigated independently in previous studies. However, the antidiabetic potential properties of fenugreek seed combined with exercise have not yet been examined. Taken together, the interaction between exercise and fenugreek is reasonable to investigate as two potential factors in the treatment of diabetes disorders. Hence, this investigation is the first study that evaluates the effect of fenugreek seed extract in combination with swimming exercise compared to glibenclamide consumption on type 2 diabetic rats.

## Materials and methods

### Experimental animals

Eighty Wistar albino male rats, weighing 200–250 g and on average 8 weeks old, were used in this study. They were housed in metal cages under standard laboratory conditions (12:12-h light–dark cycle) and were fed regular pellets and distilled water ad libitum. The room temperature was kept between 20–25°C, relative humidity 50–60%, and average illuminance 150–200 lux (daytime). All experiments procedures related to animals were approved by the Animal Care and Use Committee (ACUC), Faculty of Medicine, University Malaya (UM), Kuala Lumpur, Malaysia with ethics number: FIS/22/11/2011/FD(R).

### Diabetes induction

After a week of acclimatizing, the animals received an intraperitoneal (IP) injection (55 mg/kg) of streptozotocin (STZ, Sigma–Aldrich, St. Louis, MO, USA) for 5 consecutive days to induce diabetes. Distilled sodium citrate buffer (0.1 M, pH 4.5) was used to prepare the injection solution. Rats were fasted 14 h before injection. Hyperglycemic animals with fasting blood glucose (FBS) more than 300 mg/dL were considered diabetic. Blood samples were collected from the tail vein to measure the rats’ blood glucose using a glucometer (Bionime GM300). After a week of diabetes induction, the animals were transferred to conduct the treatment and experimental parts.

### Extraction of aqueous plant material

Fenugreek seed were purchased from the local herbal market (Tehran, Iran), then cleaned, dried, and finely powdered in a grinding machine. Powdered fenugreek seed (1.5 kg) was boiled in 1.5 L distilled water for 30 min. Then, the decoction prepared was filtered through a 0.2-mm filter paper and was concentrated by rotary evaporator to isolate the pure fenugreek seed extract. The resulting extract was concentrated under reduced pressure and stored at −20°C until use. This research followed natural remedies as organic or traditional recipe to ensure that they are safe.

### Acute toxicity study

In order to demonstrate the safety dosage of the plant extract, acute toxicity of the plant was carried out in adult male and female Wistar albino rats. The experiment was conducted according to the guidelines given in Organization for Economic Co-operation and Development (OECD) No. 420. Twenty male and female Wistar albino rats were assigned evenly into two groups and administered orally 8 g/kg of the extract in a single dose, using intragastric tubes or distilled water as a vehicle. The rats were fasted overnight prior to the dosing (free access to water), and food was withheld for another 3–4 h after dosing. They were observed for 30 min and 2, 4, 8, 24, and 48 h following the administration to monitor any onset of clinical or toxicological symptoms. Any signs of toxicity, behavioral changes, and mortality were recorded over a period of 2 weeks. The rats were sacrificed on day 15. Following sacrificing the rats, the blood was collected for serum biochemical analysis for signs of toxicity.

### Animals study design

The rats were randomly divided into the following eight groups, each with 10 animals: Groups 1) diabetic control (C), 2) fenugreek extract 0.8 g/kg (F1), 3) fenugreek seed extract 1.6 g/kg (F2), 4) swimming training (S), 5) swimming training+fenugreek extract 0.8 g/kg (SF1), 6) swimming training+fenugreek extract 1.6 g/kg (SF2), 7) glibenclamide (G), and 8) swimming training+glibenclamide (SG). Diabetic control (C) received normal saline (5 mL/kg). SG and G groups received glibenclamide by 0.5 mg/kg (29). The rats were orally administered either fenugreek (high and low dose), glibenclamide, or saline once a day with the respect treatment using intragastric tubes daily for 6 weeks. They were weighed weekly during the study. At the end of 6 weeks of treatment and after 12 h of fasting, rats were sacrificed by cardiac puncture, and blood samples were obtained from the heart. The blood was centrifuged for 5 min at 3,000 rpm in 4°C, whereupon the plasma was separated carefully and stored at −80°C for further analysis.

### Endurance training program

The protocol of swimming exercise was conducted in two phases: adaptation and training. The adaptation phase consisted of the first week of training. At the first day, the rats were warmed up in a round plastic tank (140×60×45 cm) for 10 min at water temperature of 25–30°C. The period of exercise was extended 10 min every day until the rats were able to swim for 60 min. The training phase was consisted of 60-min/day swimming, 5 days/week for a total of 6 weeks. Swimming exercise was selected because it does not cause foot damages and is physically less traumatic for the rats.

### Blood sampling and determination of biochemical variables

Animals from the trained and sedentary treated groups were euthanized at the end of 6 weeks of swimming training and gavage procedure. After sacrificing the rat, the whole blood was collected in a separator tube, allowed to clot at room temperature for 30 min. The clotted blood was then centrifuge at 3,000 g for 15 min. Serum samples were then aliquot and stored at −20°C. All scarifications were performed at the same period. Serum glucose and total cholesterol were measured using an enzymatic glucose oxidase assay with a digital spectrophotometer (Spectronic, San Diego, CA, US), whereas fasting insulin level was measured using ELISA (enzyme-linked immunosorbent assay) kit (Gloria, Heidelberg, Germany). Insulin resistance was obtained using the previously validated homeostasis model assessment of insulin resistance (HOMA-IR); HOMA-IR=fasting glucose (mmol/L)×fasting insulin (µU/mL)/22.5 (30). Using this method, higher HOMA indices denote a higher systemic resistance to insulin-mediated glucose disposal. Adiponectin and leptin levels were measured by using ELISA Kit (Abcam-Cambridge, UK). LDL was calculated using the Friedewald equation (31), and VLDL was calculated using TG/5 formula. To measure HDL, HDL-C diagnosis kits (abcam-ab65390, Abcam-Cambridge, UK) were used following the photometric method.

### Statistical analyses

Shapiro–Wilk test was conducted to determine if the data were normally distributed. All data were presented as mean±standard deviation. Analysis of variance (ANOVA) was performed using SPSS version 18 and applied to the measures of central tendency and dispersion. Two-way ANOVA was used for comparing the effect of exercise and fenugreek extract and the combination of them on diabetic parameters. A Tukey post hoc analysis was used to check for significant differences among the main effects of each dependent variable. Statistical significance was considered when *p* value was less than 0.05.

## Results

### Acute toxicity study

To determine the acute toxicity of fenugreek seed extract, the animals were orally administered with a single dosage of 8 g/kg fenugreek and distilled water as a vehicle. Their health conditions were screened throughout the experiment, and the serum biochemical parameters were evaluated. Table 1 presents the results of biochemical parameter of liver and renal function test in acute toxicity study. Biochemical parameters of renal and liver function test compare to normal range showed no significant differences between the treated group and the control group. The results from this study did not show any abnormalities in serum biochemical indicators for the functions of kidney and liver. All animals were healthy without any sign of toxicity at the tested dose, and no mortality was recorded during the experiment.

**Table 1:** The effect of *Trigonella foenum-graecum* (fenugreek) seed extract on biochemical parameters of renal and liver function tests in an acute toxicity study in animals

Biochemical analysis	Control group (vehicle, distilled water)	Treated group (8 g/kg of fenugreek extract)	Normal range
Renal function			
Sodium (mmol/L)	138.71±0.67	139.12±0.73	139.01±0.39
Potassium (mmol/L)	6.41±0.25	5.84±0.29	6.25±0.57
Urea (mmol/L)	6.28±0.44	5.98±0.61	6.10±0.48
Creatinine (mmol/L)	52.11±2.94	50.98±3.13	51.78±4.11
Liver function			
lbumin (g/L)	12.15±0.53	12.16±0.59	11.92±1.87
TB (µmol/L)	2.02±0.17	1.97±0.28	2.06±1.13
CB (µmol/L)	1.06±0.21	1.11±0.24	1.15±0.82
AP (IU/L)	135.6±9.18	136.8±10.11	135.2±13.83
Globulin (g/L)	60.22±2.7	59.89±3.15	59.28±5.85
ALT (IU/L)	52.88±4.67	51.98±5.37	53.26±6.19
AST (IU/L)	153.7±5.93	154.2±8.44	152.9±3.86
GGT (IU/L)	5.03±1.12	5.27±1.16	5.05±2.01
Total protein (g/L)	75.64±3.94	73.62±4.32	74.92±6.11

TB: total bilirubin; CB: conjugated bilirubin; AP: alkaline phosphatase; ALT: alanine aminotransferase; AST: aspartate aminotransferase; GGT: G-glutamyl transferase. Values are expressed as mean±S.E.M. There are no significant differences among groups. Significant value at *p*<0.05.

### Animal body weight

The effects of fenugreek seed or in combination with swimming exercise on rat weight within 6 weeks are presented in Table 2. All groups lost their body mass while some groups lost weight more readily, but this interaction was not significant until the third week. Furthermore, over the 6 weeks, there was a significant group week interaction.

**Table 2:** The effect of fenugreek seed administration, swimming training, and glibenclamide for 6 weeks on body weight

Groups	Week 1 (g)	Week 2 (g)	Week 3 (g)	Week 4 (g)	Week 5 (g)	Week 6 (g)
C	204.75±19.94	196±19.24	184.87±20.43	173.12±19.94	158±17.89	143.75±16.17
F1	197.5±15.56	191.5±14.02	181.9±12.44	176.3±8.78	161.3±7.05	150.1±9.90
F2	196.66±11.45	201.88±37.32	184.55±11.29	177.11±13.12	170.66±17.48	165.33±23.08
SF1	205.9±7.35	201.72±7.45	195±7.19	186.81±8.91	181.72±9.8	177.72±11.55
SF2	204.12±7.39	200.87±7.37	195.87±7.6	189.25±8.76	184±9.68	178.25±11.94
S	215.11±14.95	203.33±13.23	199.66±10.95	185.33±9.83	170.33±9.38	158.22±11.07
G	200.2±12.75	195.3±12.03	186.9±11.98	178.5±13.78	172.6±14.84	167.1±17.45
SG	205.3±18.73	200.6±17.41	193.9±16.33	187.3±15.90	180.6±17.03	174.3±18.17

Values are presented as mean±S.D. C: diabetic control; F1: fenugreek seed extract 0.8 g/kg; F2: fenugreek seed extract 1.6 g/kg; SF1: swimming training + fenugreek seed extract 0.8 g/kg; SF2: swimming training + fenugreek seed extract 1.6 g/kg; S: swimming training; G: glibenclamide; SG: swimming training + glibenclamide.

### Biochemical analysis

The fenugreek seed and swimming effects on blood biochemical variable changes in diabetic groups are presented in Table 3.

**Table 3:** The effect of fenugreek seed administration, swimming training, and glibenclamide for 6 weeks on diabetic parameters

Diabetic groups	Diabetic control (C)	Fenugreek extract 0.8 g/kg (F1)	Fenugreek seed extract 1.6 g/kg (F2)	Swimming training (S)	Swimming training+fenugreek extract 0.8 g/kg (SF1)	Swimming training+fenugreek extract 1.6 g/kg (SF2)	Glibenclamide (G)	Swimming training+glibenclamide (SG)
Glucose mmol/L	31.8±2.55#	28.7±1.62* #	25.8±1.82*	24.3±1.4 *	21.7±3.61* #	24.0±2.31*	24.2±3.05*	20.7±1.95* #
Insulin µU/ml	9.1±0.24#	8.6±0.56* #	7.8±0.35*	8.4±0.61*	8.3±0.46*	7.2±0.51*	7.3±0.21*	7.1±0.32*
HOMA-IR	12.9±0.45#	9.9±0.28*	9.9±0.39*	9.1±0.62*	8.0±0.84*	7.7±0.64*	7.9±0.76*	6.5±0.37*
Cholesterol mg/dl	119.3±2.8#	92.8±3.12* #	87.3±1.24*	86.6±2.59*	83.5±4.12*	81.3±5.33*	77.4±6.03*	75.8±5.17*
Triglyceride mg/dl	198.4±8.9#	139.8±7.3* #	122.5±9.4*	149.6±6.1*	124.5±4.8*	118.7±5.3*	112.5±16.8*	109.8±13.2*
HDL	18.4±2.1#	21.7±2.5* #	24.8±3.2*	20.3±4.6* #	25.0±5.2*	28.7±3.5*	27.3±7.4*	29.2±6.3*
LDL mg/dl	69.5±4.1#	39.3±6.2*	36.8±3.9*	39.4±5.7*	35.8±7.2*	33.7±5.1*	31.8±4.9*	29.6±6.8*
VLDL mg/dl	42.7±2.8#	32.4±1.9* #	29.2±3.6	36.6±4.1*	30.6±3.5	27.5±4.4	26.8±2.3*	24.9±3.5*
Leptin ng/mL	3.58±0.5#	3.39±0.7#	3.02±0.3*	2.88±0.8*	2.97±0.7*	2.81±0.5*	2.46±0.8*	2.32±0.47*
Adiponectin	6.49±1.0#	6.67±0.5#	6.81±0.6*	6.58±0.9*	6.72±0.8*	7.07±0.6*	7.21±1.2*	7.28±0.8*

Values are presented as mean±S.D (*n*=6). C: diabetic control; F1: fenugreek seed extract 0.8 g/kg; F2: fenugreek seed extract 1.6 g/kg; SF1: swimming training+fenugreek seed extract 0.8 g/kg; SF2: swimming training+fenugreek seed extract 1.6 g/kg; S: swimming training; G: glibenclamide; SG: swimming training+glibenclamide;

* Significant decrease.

* Shows statistically significant compared with diabetic control

# presents statistically significant compared with glibenclamide (*p*<0.05).

#### Glucose

The plasma glucose concentration levels in the F1, F2, S, SF1, SF2, G, and SG groups were significantly decreased compared to the C group (*p*<0.05), and among the groups, SF1 and SF2 groups exhibited the most reduction.

#### Insulin

Significant differences exist among the diabetic groups in terms of insulin concentration level. However, the insulin level insignificantly decreased in the all treated and in combination with swimming compare to control.

#### HOMA-IR

Significant differences of HOMA-IR reduction levels were observed in all groups compared with the control group.

#### Cholesterol and TG

The concentration levels of cholesterol and TG were significantly lower in the all treated and swimming diabetic subgroups compared to control.

#### HDL

The HDL concentration levels in all the treated groups and swimming diabetic subgroups significantly increased compared to control.

#### LDL and VLDL

The concentration levels of LDL and VLDL in all the treated groups and swimming diabetic subgroups significantly decreased compared to control.

#### Hormones leptin and adiponectin levels

Plasma leptin concentrations were significantly lower in all groups compared to the control group. Significant differences in adiponectin concentration induction levels were observed in all groups compared to the control group.

## Discussion

In general, the present study revealed that diabetic rats treated by fenugreek seed and glibenclamide could improve the glycemic and lipoprofile control. However, the high-dose treatment with fenugreek seed extract showed more effective compared to the low-dose treatment. This improvement was more highlighted in the swimming training with fenugreek treated groups compared to control or glibenclamide treated group. Although the case of total recovery of diabetes has never been reported (32, 33) but could be improved by chemical or biochemical agents. It has been reported that there are more than 1,000 plant species being used for the treatment of this silent disease (34). Fenugreek seed, a rich source of bioactive antioxidant substances, is commonly used as an important ingredient in daily food preparations, and phenolic compounds are used as herbal formulations (12, 19). We have speculated that consumption of fenugreek seeds combined with swimming training strongly improves diabetic parameters.

The primary deduced data from the acute toxicity test was used to choose the safe doses and identify the toxicity effects. The results obtained from acute toxicity study showed no toxicity effect of fenugreek seed extract at the tested dose. The findings of this study agreed with previously reported studies on fenugreek leaf extract or ethanolic extract of fenugreek seed (35, 36). Our findings have shown that rats of all groups had lost their body weight, while this reduction was significantly observed in the diabetic control croup. The animals in the treated and trained groups have also showed slight loss in body mass. Furthermore, body weight loss and fluctuations in body composition can be the reasons for FBS reduction, which is caused by metabolic marker improvement. FBS level in STZ-induced diabetic rat was significantly decreased compared with the control group, fenugreek seeds extract treated group, and the glibenclamide group, and this hypoglycemic effect was more highlighted when these treatments were combined with swimming training. The results from present study showed promising and dose-dependent antihyperglycemic effects of the extract in animal model of diabetes mellitus. Exercise has been suggested as a potential strategy to improve glycemic control and help with weight control. In previous studies, significant reductions of serum glucose and lipid profile in diabetic patients have been reported through exercise (37, 38).

Furthermore, muscle contraction under metabolic stress conditions cause a rapid depletion of glycogen and change the binding of various proteins linked to glycogen. This function requires a considerable amount of muscle fibers to refill their carbohydrate reserves. Reconstruction of glycogen storage with changes in molecular structure and non-esterified fatty acids concentration reduction contributes to the insulin sensitivity regulation in skeletal muscles after exercise training (39). Therefore, an increase in lean body mass after resistance exercise may be an important mediator in the improvement of glycemic control. Probably because of resistance training, the muscle mass even without altering the intrinsic capacity of the muscle to respond to insulin improves glucose disposal (38).

Our findings further revealed that 6 weeks of resistance training with fenugreek seed extract had significant effects on insulin and insulin resistance reduction in diabetic rats. This improvement was more pronounced in the group with swimming training and extract treatment, the effects of which were comparable with that of glibenclamide. A study conducted by Kannappan and Anuradha (40) have suggested an improved insulin signaling and sensitivity in response to fenugreek seed extract consumption through promoting the cellular actions of insulin (40). The positive effects of swimming exercise on insulin resistance improvement can be reached through insulin receptor enhancement in muscle cells or an increase in the number of glucose transporter proteins in the skeletal muscle cells. The insulin-resistant in type 2 diabetics is mainly associated with the defect in glucose uptake and the dysregulation of glucose transporter-4 (GLUT-4) protein (41). GLUT4 is insulin-responsive and benefits glucose transportation through muscle contraction and insulin in the muscles. Hence, the increase in total muscle mass because of resistance exercise leads to an increased glucose uptake through insulin mediation (37). Therefore, fenugreek seeds may stimulate and involve with the regulation of GLUT 4 signaling pathways to modulate insulin.

We have also found that plasma leptin concentrations were significantly decreased, whereas adiponectin concentrations increased when treated with fenugreek seeds specifically in groups that received extract in combination with exercise. Hypoadiponectinemia leads to greater insulin resistance and increased risk of type 2 diabetes (42). Adiponectin enhancement by physical activity has also been reported (43, 44), the results of which were in line with previous studies’ reports. However, the studies on the effect of fenugreek seed constituents on adiponectin level are very limited. Unlike most other adipokines, adiponectin levels in obese individuals are lower compared with those in lean people (45–47). Nevertheless, the fragmentation of adiponectin molecules is likely to be elevated in obese patients (48). Furthermore, elevated circulating leptin concentrations are also associated with increased body fatness, and increased body fatness is strongly associated with insulin insensitivity. Bouloumie et al. reported that leptin applies atherogenic and angiogenic properties through the generation of oxidative stress in endothelial cells (49, 50); another study recently demonstrated a vascular calcifying effect of leptin (51). Meanwhile, obese people with high adipose tissue are required to have more vascular bed to maintain blood circulation (52). Therefore, this adaptation might conversely improve arteriosclerosis in the long run. However, further profound studies are needed to investigate the effects of the combination of endurance training and fenugreek seed extract at molecular level on leptin and adiponectin.

The present study showed that consumption of fenugreek seed extract had a significant reduction effect on cholesterol, TG, HDL, LDL, and VLDL. Studies have proved the positive influence of fenugreek seeds on improved lipid profiles (53). Nonetheless, the reason for this reduction is not entirely clear and may be because of the changes occurring in the metabolism of triglycerides and lipoproteins during exercise (54). Another study demonstrated that exercise increased plasma LPL and hepatic lipase which mediated TG clearance (55). In the present study, an obvious decrease was observed in the TG, VLDL, and LDL (56–58) levels after 6 weeks of training, which is supported by previous studies. In view of the positive changes to lipid profile, physical activity protocols are recommended to increase lipolysis, decrease TG, and improve the ratio of oxidant to antioxidant, and to change LDL-C synthesis or plasma LDL-C elimination by tissue. Some investigations have suggested that changes in lipid profile by practice are possibly associated with a change in fat mass (59). The results of the lipid profile suggest that physical activity may have a crosstalk with fenugreek seeds constituents in lipid metabolism of diabetic disorders.

The treatment using the extract in combination with swimming training demonstrated hypoglycemic activity against STZ-induced hyperglycemia rats. The results from this study were in line with previously studies (4, 60). Studies have strongly supported the benefits of exercise training in the prevention and treatment of insulin resistance, impaired glucose homeostasis, and NIDDM (4, 5, 20, 60). Our data showed that, in diabetic rats, exercise could modulate circulating leptin concentrations. The lack of treatment effect in leptin modulation may simply be because of the fact that induced diabetes produced such weight loss that, apart from the glibenclamide group, this overwhelmed any further subtle effects of each treatment. A number of studies have investigated the effect of swimming training on glucose and insulin levels in diabetic rats. Mohammad et al. (61) reported that swimming training had beneficial effects on insulin regulation in diabetic rats. Of the various types of physical training, swimming exercise especially seems useful for treating metabolic diseases associated to obesity and obesity-related diabetes (62, 63) because it is weight bearing and minimizes injury to joints.

Some mechanisms by which glycemic control is improved with exercise training include improved contraction-mediated glucose uptake by muscle through metabolic demands and increased muscle blood flow, increased GLUT-4 protein content, and increased insulin-mediated GLUT-4 intramyocellular translocation. Therefore physical activity may potentially benefit type 1 diabetics, insulin-resistant, and those with type 2 diabetes. The improved responsiveness to insulin induction by doing swimming in rat skeletal muscle may result relatively from modulation of insulin signaling pathway at different levels of molecular (64). In particular, the IRS/IP3- kinase pathway may involve in the glucose transport activation, muscle glycogen synthesis, thus an increase in this association in trained animals or human muscle may have a crucial role in insulin responsiveness (65).

## Conclusions

To sum up, the present study showed that treatment using fenugreek seed extract in combination with swimming training have a strong therapeutic effect on diabetic parameters in STZ- induced diabetic rats in a dose-dependent manner. Thus, with the benefits of exercise and concurrent fenugreek seed consumption on reducing health risk factors, diabetic patients are advised to exploit this combination of factors to control and manage their disease. Our study provides a key insight into diabetes, but significant evidences for the benefits of the combined factors have not been obtained. However, further investigations are warranted to identify the proper dose and role of specific compounds of fenugreek seed on diabetic parameters at the molecular level.
